# Effects of Green Rice Husk Dietary Fiber and Hydrocolloids on the Physicochemical, Structural, Bioactive, and Sensory Properties of Gummy Products

**DOI:** 10.3390/foods15071114

**Published:** 2026-03-24

**Authors:** Tipaukson Chaikwang, Hua Li, Sirithon Siriamornpun

**Affiliations:** 1 Department of Food Technology and Nutrition, Faculty of Technology, Mahasarakham University, Kantarawichai, Maha Sarakham 44150, Thailand; 67010853502@msu.ac.th; 2 Department of Cuisine and Nutrition, Yangzhou University, Yangzhou 225127, China; lihua216@yzu.edu.cn; 3 Key Laboratory of Chinese Cuisine Intangible Cultural Heritage Technology Inheritance, Ministry of Culture and Tourism, Yangzhou 225127, China; 4 Research Unit of Thai Food Innovation (TFI), Mahasarakham University, Kantarawichai, Maha Sarakham 44150, Thailand

**Keywords:** rice by-product, applications, food industry, antioxidant activity, textural properties

## Abstract

Green rice husk dietary fiber (GHDF) is an underutilized agricultural by-product with promising potential for applications in the food industry. This study investigated the effects of incorporating dietary fiber from GHDF at 1%, 3%, and 5% together with different hydrocolloids, including xanthan gum (XG), carrageenan (CC), and guar gum (GG), on the physical and chemical, textural properties, and consumer acceptance of gummy products. The results showed that adding more GHDF increased the nutritional value of the gummies, with total dietary fiber ranging from 1.01 to 5.02 g per 100 g of product. FTIR results also showed that fiber from green rice husk was present in the gummies. The combined addition of GHDF and hydrocolloids also affected the internal gel structure of the products. This interaction made the gel structure stronger, resulting in firmer gummies with greater hardness, gumminess, and chewiness. In addition, higher GHDF levels contributed to reduced syneresis. Among the hydrocolloids tested, xanthan gum produced the strongest gel, while the formulation with 3% GG received the highest consumer liking scores. These results suggest that GHDF could be used as a useful ingredient to develop food products with higher nutritional value and better use of agricultural by-products.

## 1. Introduction

Gummy products are widely consumed snack foods due to their ease of consumption and suitability for consumers of all ages [1]. In recent years, increasing attention has been given to the fortification of gummy products with health-promoting ingredients as an alternative functional food form. These ingredients include vitamins, minerals, proteins, lipids, and dietary fiber, the last of which has received increasing attention because of its reported health benefits [2].

Dietary fiber is found in two main types: soluble dietary fiber (SDF) and insoluble dietary fiber (IDF). Numerous studies indicate that increased fiber consumption can reduce the risk of heart disease, diabetes, and cancer [3]. IDF is the most common form of dietary fiber, and it can make up as much as 90% of the total dietary fiber in some plants. The main elements of IDF are cellulose, hemicellulose, and lignin. These provide it with a porous structure and a high level of crystallinity [4]. The structural and functional properties of IDF can affect the physical properties, sensory quality and stability of food products into which it is incorporated.

Rice husk is a major by-product of the rice milling process, accounting for approximately 20% of the total grain weight. Its chemical composition primarily comprises cellulose (34.4%), hemicellulose (24.3%), lignin (19.2%), ash (18.85%), and other components (3.25%) [5], indicating that rice husk is a promising source of insoluble dietary fiber. However, it remains largely underutilized and is commonly discarded or used in low-value applications. Previous studies have demonstrated the feasibility of dietary fiber extraction from rice husk and emphasized its potential as a value-added material from the rice milling industry [6]. Moreover, rice husk has been reported to contain bioactive compounds with functional properties, including antioxidant activity, which may confer health benefits [7]. Recent research has highlighted the influence of rice growth stages on dietary fiber quality and bioactive composition. Chaikwang et al. [8] noted that dietary fiber extracted from green rice husks collected 22–28 days after flowering displayed considerable levels of cellulose, beta-glucan, and bioactive compounds, including phytosterols. These findings are consistent with earlier reports indicating higher abundance and diversity of bioactive compounds at earlier rice growth stages [9]. Nevertheless, systematic studies on the utilization of green rice husk as a functional dietary fiber source, particularly regarding its physicochemical properties and applications in food systems, remain limited. Therefore, green rice husk dietary fiber (GHDF) represents a promising alternative raw material that warrants further investigation for the development of functional and health-oriented food products.

Hydrocolloids are complicated chemicals that can make food systems more stable, make liquids thicker, and make gels. They change the texture and how much people like the food [10]. Hydrocolloids are also used to improve the texture of gummy foods [11]. Guar gum (GG), xanthan gum (XG), and carrageenan (CC) are all hydrocolloids that are commonly used as ingredients in sticky food formulations to give them a more consistent texture and structure. Guar gum is known for being quite thick, being able to hold water well, and keeping things stable [12]. Xanthan gum is a common anionic hydrocolloid that is thick and has a gel-like shape because it has a strong core structure and charged side chains [13,14]. Carrageenan is a negatively charged polysaccharide from seaweed whose galactose-based structure supports elastic texture and gel stability [10]. Although hydrocolloids are effective in improving the structure and texture of gummy products, the incorporation of insoluble dietary fiber may influence the stability of the gel system and the resulting textural properties. However, insoluble dietary fiber derived from green rice husks has attracted interest as a by-product of the rice milling process with potential nutritional value. Despite these advantages, the application of green rice husk dietary fiber (GHDF) in food products, particularly in gummy formulations, remains relatively limited in the literature.

Therefore, the objective of this study was to develop formulations and processing conditions for gummy products fortified with dietary fiber extracted from green rice husk in combination with different hydrocolloids, and to evaluate their physicochemical properties, chemical properties, textural characteristics, rheological behavior, bioactive compound content, and sensory acceptance. The findings are expected to provide baseline information supporting the potential use of dietary fiber from green rice husk, an agricultural by-product of rice milling, as a functional ingredient in health-oriented food development, with possible future industrial applications. This approach is generally aligned with concepts of by-product valorization and sustainable development.

## 2. Materials and Methods

### 2.1. Materials and Reagents

Green rice husk dietary fiber (GHDF) was obtained from our previously published study by Chaikwang et al. [8]. The GHDF used in this study had a fine particle size ranging from 125 to 100 μm. Sugar and glucose syrup were purchased from the local market. Gelatin, citric acid, carrageenan (CC), guar gum (GG), and xanthan gum (XG) were obtained from Krungthepchemi Co., Ltd. (Bangkok, Thailand). All chemical reagents used for analytical procedures were sourced from Sigma-Aldrich (St. Louis, MO, USA).

### 2.2. Gummy Preparation

The samples were prepared according to the method described by Delgado-Durán et al. [15], with slight modifications. The ingredients used are listed in Table 1. The dry ingredients were first mixed thoroughly, followed by the gradual addition of the liquid ingredients while stirring continuously to ensure homogeneity. The mixture was heated to 60 °C for 60 s to allow complete melting of the solid components and formation of a homogeneous phase, followed by molding and storage at 4 °C for further analysis.

### 2.3. Color Parameters, Water Activity (a_w_), and pH Value

The color of the gummies was measured with a chromameter (Minolta, Model CR-400, Tokyo, Japan). Water activity (a_w_) was determined at 25 °C using a water activity meter (AquaLab PRE, Pullman, WA, USA). The pH was measured with a pH meter (PB-10, Sartorius, Göttingen, Germany) at room temperature.

### 2.4. Syneresis

Syneresis was analyzed based on water loss from the gel using a modified version of the method of Figueroa & Genovese [16], with an analysis period of 7 days. The percentage of syneresis was calculated using the following equation.

(1) Syneresis % = (m_i_ − m_f_)/m_i_

where m_i_ and m_f_ are the initial and final masses of the gummy, respectively.

### 2.5. Texture Profile Analysis (TPA)

The textural properties of the samples were measured using a TA-XT Plus texture analyzer (Stable Micro Systems, Surrey, UK), following the method described by Tarahi et al. [17] with minor modifications. The analysis was performed using Force Recorder (ZT-RP) software version 6.2 with a 40 mm diameter compression probe, a pre-test speed of 2 mm/s, a post-test speed of 10 mm/s, a test distance of 2 mm from the platform, and a trigger force of 0.05 N.

### 2.6. Rheological Properties

The rheological properties of the gummy samples were measured using a rheometer (HAAKE MARS iQ Air, Thermo Fisher Scientific, Waltham, MA, USA) equipped with a parallel plate geometry (P35/Ti-02180932). The plate gap was set to 2.5 mm, and all measurements were conducted at 25 °C. Oscillatory frequency sweep tests were performed within the linear viscoelastic region following the method of Latrofa et al. [18] with minor modifications. The frequency was varied from 0.1 to 10 Hz at a constant strain of 1%.

### 2.7. Proximate Composition

The proximate composition of the samples (moisture, protein, ash, lipid, and fiber) was analyzed using standard AOAC methods [19], specifically methods 969.19, 920.177, 900.02, 920.176, and 985.29. Total carbohydrate content was calculated by difference, and all values were expressed as g/100 g fresh weight (FW).

### 2.8. FTIR Analysis

FT-IR spectroscopy was employed to identify the functional groups present in the samples. Measurements were performed using an INVENIO S/LUMOS II spectrometer (Bruker, Fällanden, Switzerland) in transmission mode over the wavenumber range of 4000–400 cm^−1^ with a resolution of 4 cm^−1^.

### 2.9. Antioxidant Activities and Bioactive Compounds

#### 2.9.1. Total Phenol Content (TPC) and Total Flavonoid Content (TFC)

The TPC and TFC were determined according to the method described by Raksakantong et al. [20], with slight modifications. The absorbance was measured at 725 nm for TPC and 510 nm for TFC using a UV–visible spectrophotometer (UV-1700, Shimadzu, Tokyo, Japan). TPC was expressed as mg of gallic acid equivalents per 100 g of fresh weight (mg GAE/100 g FW), while TFC was reported as mg of quercetin equivalents per 100 g of fresh weight (mg QE/100 g FW).

#### 2.9.2. Determination of Antioxidant Activity

The ferric reducing antioxidant power (FRAP) assay and 2,2-diphenyl-1-picrylhydrazyl (DPPH) radical scavenging assay were conducted according to the method described by Siriamornpun et al. [21]. For the FRAP assay, absorbance was measured at 593 nm, and the results were expressed as mg of ferrous sulfate equivalents per 100 g of fresh weight (mg FeSO_4_/100 g FW). For the DPPH assay, absorbance was measured at 510 nm using a spectrophotometer, and the antioxidant activity was reported as mg of L-ascorbic acid equivalents per 100 g of fresh weight (mg AA/100 g FW).

### 2.10. Sensory Analysis

The sensory acceptance test was conducted following the method of Seepua et al. [22] and Stokes et al. [23], with slight modifications. Gummy samples fortified with green rice husk dietary fiber were evaluated for appearance, color, aroma, taste, texture, and overall liking by 30 untrained panelists (aged 18–40 years). All participants had no known allergies to the ingredients used in the samples and were familiar with gummy products. Twelve gummy formulations were prepared using molds of identical size (1 × 1 cm). To minimize sensory fatigue, the evaluation was divided into three sessions, with four samples evaluated per session. Panelists rated their liking using a nine-point hedonic scale (1 = dislike extremely, 9 = like extremely). During the evaluation, panelists were instructed to cleanse their palate between samples using deionized drinking water provided. Each evaluation session lasted approximately 30 min, followed by a 15 min break between sessions to reduce panelist fatigue. Sensory testing was conducted between 09:00 and 15:00. This study was conducted in accordance with ethical guidelines approved by the Human Research Ethics Committee of Mahasarakham University (Approval No. 430-405/2568, approval date: 27 June 2025).

### 2.11. Statistical Analysis

The results represent the mean ± SD obtained from at least three independent trials. The data were subjected to one-way ANOVA followed by Duncan’s multiple range test. Significant differences were determined at *p* < 0.05 by IBM SPSS Statistical Software version 17.0.

## 3. Results

In the experimental design, our present study focused on evaluating different levels of GHDF (1%, 3%, and 5%) in combination with different types of hydrocolloids. Since GHDF mainly consists of insoluble dietary fiber, its incorporation into the gummy formulation may lead to sedimentation of fiber particles at the bottom of the product. Therefore, hydrocolloids were incorporated into the formulation to help improve the stability of the system as well as the textural properties of the gummies. Accordingly, the study aimed to evaluate the effects of different GHDF levels in combination with hydrocolloid types on the properties of gummy products, in comparison with formulations without hydrocolloid addition. Therefore, a plain control gummy lacking both dietary fiber and hydrocolloid was not included in the formulation design of this study. Similar research has also been conducted on jelly products made from watermelon rind powder [17].

### 3.1. Color Parameters and Appearance

**Figure 1:**
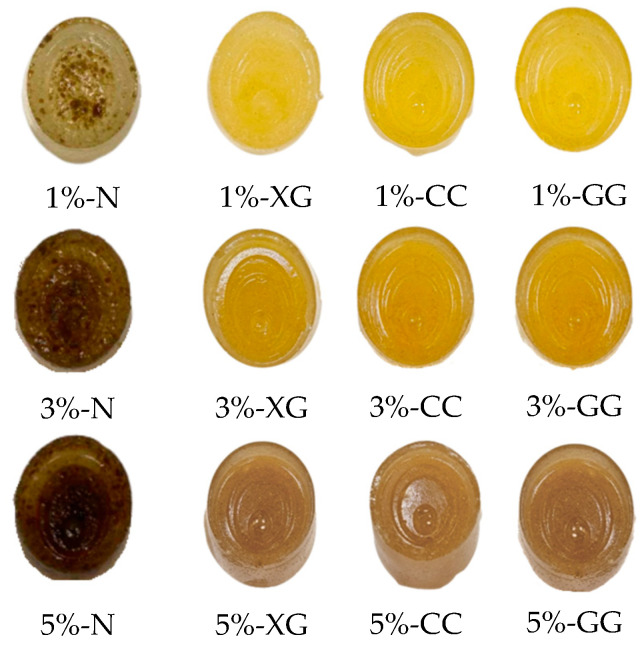
Appearance of green rice husk gummy fibers. The values 1%, 3%, and 5% represent the levels of added green rice husk dietary fiber (GDHF). N = no hydrocolloids added; XG = xanthan gum; CC = carrageenan; GG = guar gum.

Color is an important visual quality indicator of things, influencing consumer perception and purchasing decisions [17]. Table 2 presents the color values of gummy samples fortified with green rice husk dietary fiber. Both the GHDF concentration and the type of hydrocolloid had a significant effect on the color. The L* values ranged from 31.20 to 46.06, a* values from 0.05 to 1.59, and b* values from 6.50 to 11.03. Increasing the dietary fiber concentration resulted in decreases in L*, a*, and b*, indicating a darker overall appearance. This trend aligns with the findings of Figueroa & Genovese [16], who found that fruit jellies with higher fiber content exhibited similar changes, and of Gok et al. [24], who noted slight darkening in products containing wheat fiber, likely due to physical interference of fiber particles with light reflection within the gel matrix. In addition, the type of hydrocolloid played a significant role in determining the color and uniformity of the gummy products. Figure 1 shows that samples without hydrocolloid had fibers settling to the bottom of the container and uneven color. A frequent challenge arises in systems that include insoluble dietary fiber [25]. The addition of hydrocolloids enhanced product uniformity by increasing system viscosity and stabilizing the dispersion of fiber particles [10]. Consequently, the optimization of both the hydrocolloid selection and the dietary fiber concentration is crucial for regulating color characteristics and achieving an appealing product appearance, thereby influencing consumer acceptance.

### 3.2. Water Activity (a_w_) and pH Value

Water activity (a_w_) and pH are important parameters related to the safety and shelf life of food products [26]. Table 2 shows the a_w_ values of gummy products made with different amounts of green rice husk dietary fiber (GHDF) and different hydrocolloids. The a_w_ values range from 0.87 to 0.89, reflecting relatively high levels of free water in the products. Although these relatively high a_w_ values may increase the susceptibility of the product to microbial growth during storage, they fall within the range reported for similar gel-like confectionery products, such as watermelon rind gummies (0.710–0.990) reported by Krajewska et al. [27] and orange peel jelly gummy products (0.928–0.935) reported by Lucas-González et al. [26] This difference may be related to the contribution of dietary fiber and hydrocolloids in retaining water within the product matrix [24]. Increasing the amount of dietary fiber from green rice husk tends to lower the a_w_ value. This observation is consistent with the findings of Krajewska et al. [27], who reported that adding dietary fiber from watermelon to gummy products helps lower the a_w_ value because the fiber can hold onto water in the gel structure. The fiber’s ability to hold onto water may assist in lowering the amount of free water in the system. This might make the product more stable and lower the risk of microbial growth.

The product has a pH of about 4.87, which is in the range of pH levels that are common for gummy products (pH 3–5) [26]. There were no major variations in pH between formulations with different amounts of dietary fiber and hydrocolloids. Hasani & Yazdanpanah, 2020 [28] found similar results. They said that replacing pectin with gum did not change the pH of the gel system much because these gelling agents do not have strong acidic or alkaline qualities. But the acidity of the finished product may change when different types of dietary fibers are mixed together, depending on the properties of the raw materials. Romo-Zamarrón et al. [29] noted that using pineapple peel powder lowered the pH of the gummy candy formulation, but the inclusion of papaya fiber marginally elevated the pH. Similarly Cedeño-Pinos et al. [30] reported that the incorporation of rosemary extract did not significantly affect the pH of gummy candy formulations (*p* > 0.05). Additionally, Lucas-González et al. [26] reported that adding orange peel extract to jelly candies did not significantly alter the pH, with the pH remaining in the range of 5.23 to 5.28. Therefore, the measured acidity may vary depending on the raw materials used, as well as other factors in terms of quantity and composition in the formula.

In general, the a_w_ value of gummy with green rice husk fiber is rather high. This study gives us some early information about the physical and chemical properties of gummy goods with green rice husk fiber. More research is needed to look at how storage affects the stability of these items, such as how microbes develop and how the physical and chemical properties change over time. This will help us better understand how dietary fibers and hydrocolloids affect the shelf life of these products.

### 3.3. Syneresis

Syneresis is the separation of liquid from a gel matrix after gel formation, resulting in visible water release [31]. As shown in Table 2, the syneresis of the gummy samples varied depending on the level of green rice husk dietary fiber (GHDF) and the type of hydrocolloid used, with values ranging from 0.03% to 6.44%. The results suggest that increasing the concentration of GHDF was associated with reduced liquid separation in the gummy matrix. This finding is consistent with the study of Figueroa and Genovese [16], who reported that natural dietary fibers effectively reduce liquid separation in jelly systems. In addition, previous studies have demonstrated that the incorporation of IDF enhances the water-holding capacity of gels by binding water on the fiber surface and modifying the gel structure to retain water more effectively within the system [32]. These effects may be attributed to the plant cell wall structure of dietary fiber, which is favorable for water retention within the gel network [33]. However, excessive fiber addition sometimes increased syneresis, likely due to coarse particle size or low water-holding capacity that disrupted the gel network [34].

In relation to the effect of hydrocolloids on water separation, the addition of hydrocolloids significantly reduced water separation compared to samples without hydrocolloids. Among the hydrocolloids tested, xanthan gum exhibited the most pronounced reduction in water separation, consistent with the findings of Khouryieh et al. [35], who reported that xanthan gum effectively reduces water separation in gel systems. Furthermore, Lin et al. [36] highlighted the role of hydrocolloids in reducing syneresis during the storage of milk pudding products. Hydrocolloids play a critical role in limiting syneresis by reinforcing gel networks and enhancing water retention within the system [37]. The stability of the final product may be affected by other components in the formulation, such as insoluble dietary fiber and the type of hydrocolloid used, both of which influence how water separation is reduced. Therefore, determining the optimal amount and physical and chemical properties of dietary fiber in formulations is crucial for gel stability and controlled water separation, supporting the use of GHDF in food product development.

### 3.4. Texture Profile Analysis

**Figure 2:**
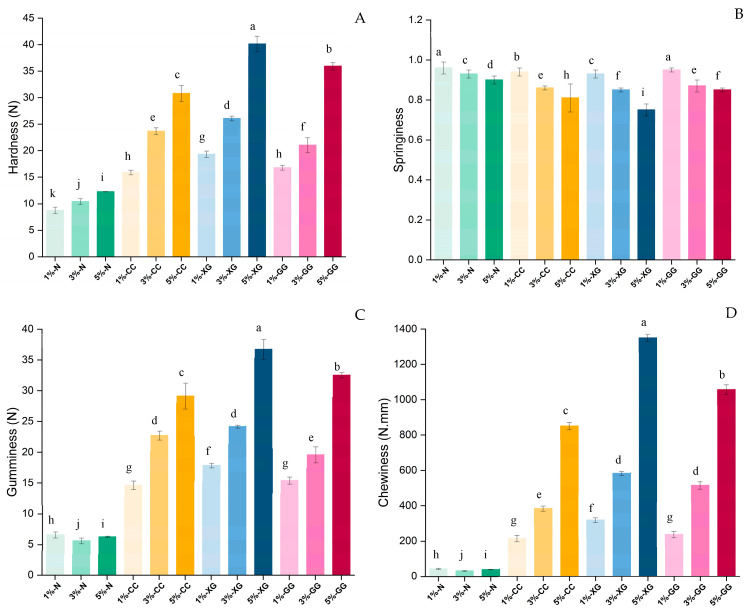
Texture profile analysis (TPA) of green rice husk gummy fibers: (**A**) hardness, (**B**) springiness, (**C**) gumminess, and (**D**) chewiness. The values 1%, 3%, and 5% represent the levels of added green rice husk dietary fiber (GDHF). N = no hydrocolloids added; XG = xanthan gum; CC = carrageenan; GG = guar gum. Superscripts ^a–k^ indicate significant differences among means within the same bar (*p* < 0.05).

Texture profile analysis (TPA) showed that both the concentration of dietary fiber from green rice husk and the type of hydrocolloid significantly affected the textural properties of gummy samples (*p* < 0.05), as shown in Figure 2. Hardness values ranged from 8.77 to 40.14 N, while springiness ranged from 75 to 96%, respectively, reflecting the integrity of the gel structure and its ability to recover after deformation. In addition, gumminess (6.27–32.51 N) and chewiness (31.68–1056.87 N) represent the overall energy required for mastication of the product. The increasing addition of GHDF, an insoluble dietary fiber derived from green rice husk, significantly increased the hardness, gumminess, and chewiness of the gummy sample. This observation is consistent with previous studies reporting that an increase in solid content, particularly insoluble dietary fiber, enhances gel density and mechanical strength in jelly and gummy systems [38,39]. This effect may be attributed to the function of insoluble dietary fiber as a filler within the gel matrix [40], which could contribute to a more compact structure and enhanced resistance to deformation [41]. However, excessive fiber addition may lead to non-uniform dispersion, which disrupts the gel network and increases brittleness, resulting in reduced springiness [24].

Regarding the type of hydrocolloid, the addition of hydrocolloids generally enhanced the strength of the gel matrix and improved overall textural integrity [42]. Xanthan gum exhibited the highest hardness, gumminess, and adhesiveness, which may be attributed to its rigid molecular conformation and the formation of a stable network through hydrogen bonding, providing high resistance to deformation [10]. Guar gum, on the other hand, had the most springiness. This is probably because its galactomannan chains are flexible, which allows them to change shape and quickly return to their original shape [43]. Compared with xanthan gum and guar gum, carrageenan exhibited less pronounced effects on textural properties. The observed differences may be partly attributed to structural variations among hydrocolloids, which could affect intermolecular interactions and the formation of the gel network within the gummy matrix [10,44]. In conclusion, the amount of insoluble dietary fiber and the type of gelling agent are important factors that influence the texture of gummy products. These results provide useful information for developing gummy recipes and support the use of dietary fiber from agricultural by-products in healthier food products.

### 3.5. Rheological Properties

**Figure 3:**
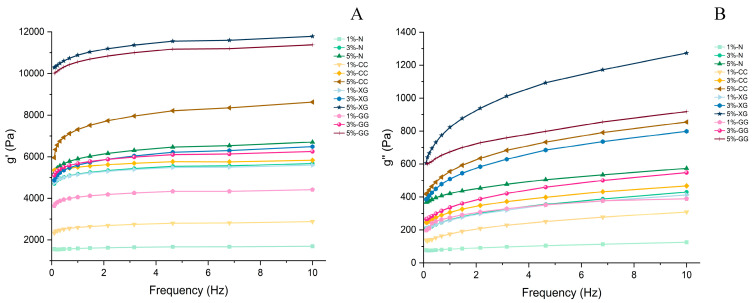
Rheological properties of GHDF: (**A**) storage modulus (G′) and (**B**) loss modulus (G″). The values 1%, 3%, and 5% represent the levels of added green rice husk dietary fiber (GDHF). N = no hydrocolloids added; XG = xanthan gum; CC = carrageenan; GG = guar gum.

A frequency sweep study was conducted in the linear viscoelastic area to assess the structural stability of the gummy systems [45]. Figure 3 shows the loss modulus (G″) and storage modulus (G′) of gummies that have varying amounts of green rice husk dietary fiber (GHDF) mixed with different types of hydrocolloids. The results showed that adding more GHDF was linked to higher G′ and G″ values. Samples with 5% fiber usually had higher moduli than samples with 3% and 1% fiber. In all of the tests, G′ was always bigger than G″. This means that the gummy matrix usually acts like a solid. This pattern is consistent with the findings of Wang & Hartel, 2022 [46], who discovered that the viscoelastic parameters change when fiber is added to gummy products. The rise in modulus may be because GHDF is largely made up of insoluble dietary fiber that fills in the structure of the gel matrix. This results in a denser network structure and restricts the mobility of polymer chains [40]. Additionally, Latrofa et al. [18] observed that the incorporation of pea fiber increased both G′ and G″, which may be related to the reinforcing effect of fiber components within the composite gel system. In addition, the type of hydrocolloid significantly influenced the viscoelastic behavior of the gummies. Formulations containing xanthan gum, guar gum, and carrageenan exhibited higher G′ and G″ values than the control without hydrocolloids, with G′ consistently exceeding G″, indicating elastic-dominant behavior. This observation is consistent with Guo et al. [47], who suggested that hydrocolloids may enhance gel network integrity and improve elastic properties, thereby affecting flow behavior. Among the tested hydrocolloids, xanthan gum showed the highest G′ values, followed by guar gum and carrageenan. These differences may be attributed to variations in molecular structure, such as the relatively rigid backbone and intermolecular interactions of xanthan gum [10], the galactomannan structure of guar gum [43], and the negatively charged sulfate groups in carrageenan [10,44]. However, the observed rheological behavior may reflect the combined effects of both hydrocolloids and dietary fiber within the composite gummy matrix [18]. Overall, the incorporation of GHDF at different levels in combination with various hydrocolloids was associated with increased viscoelastic moduli and a more pronounced solid-like behavior, suggesting a reinforcing effect within the gummy gel network.

### 3.6. Proximate Composition of Green Rice Husk Gummy

The chemical composition of the rice-husk-fiber-enriched gummies is presented in Table 3. The results indicate that varying levels of green rice husk dietary fiber (GHDF) supplementation influenced the overall nutrient composition of the products. The gummies exhibited moisture contents ranging from 44.38 to 47.72 g/100 g, protein contents of 6.23–7.43 g/100 g, carbohydrate contents of 42.81–44.21 g/100 g, total dietary fiber contents of 1.00–5.02 g/100 g, fat contents of 0.16–0.21 g/100 g, and ash contents of 0.27–0.56 g/100 g. The incorporation of GHDF at levels of 5%, 3%, and 1% resulted in a progressive increase in the overall contents of dietary fiber, protein, and ash. Compared with gummies produced from other agricultural by-products, the fiber-enriched gummies in this study showed higher fiber content than gummies made with orange peel, as reported by Lucas-González et al. [26], and those fortified with strawberry and red beet fiber, as reported by Ali et al. [48], but lower than gummies enriched with watermelon rind [49]. These variations may be attributable to the fact that the primary materials have distinct nutritional profiles and that the formulations and processing conditions are distinct [17]. In addition, the results showed that the use of different hydrocolloids induced slight variations in the chemical composition of the gummies. These effects may arise from differences in molecular structure and functional properties of the hydrocolloids, particularly their roles as thickening and gelling/stabilizing agents. Hydrocolloids increase system viscosity and reduce solvent diffusion, which may influence extraction efficiency and consequently affect the measured chemical composition [50]. This observation is consistent with the findings of Padalino et al. [51] in their research on pasta formulations, where the addition of hydrocolloids altered the chemical composition due to differences in their molecular structures. Furthermore, variations in the dispersion and distribution of hydrocolloids within the food matrix may also contribute to differences in measured chemical constituents [52]. Nevertheless, some studies have reported that hydrocolloids may not significantly influence chemical composition, depending primarily on the levels used [11].

Overall, the results show that adding GHDF can increase the nutritional value of gummy products, mainly by increasing their fiber content. These findings suggest that by-products from rice processing can be used as useful ingredients to develop healthier food products.

### 3.7. FTIR

**Figure 4:**
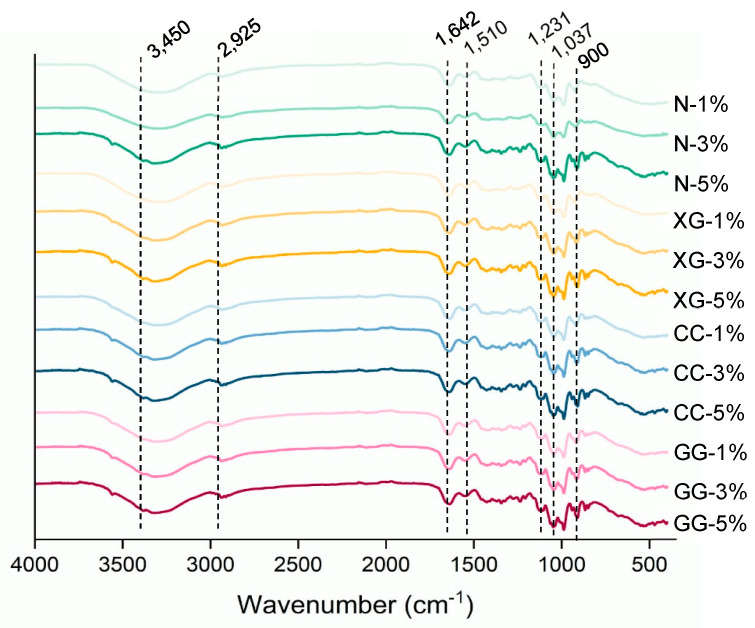
Fourier-transform infrared (FT-IR) spectra of green rice husk gummy fibers. The values 1%, 3%, and 5% represent the levels of added green rice husk dietary fiber (GDHF). N = no hydrocolloids added; XG = xanthan gum; CC = carrageenan; GG = guar gum.

FTIR spectroscopy analysis was employed to characterize the functional groups present in the gummy samples fortified with green rice husk dietary fiber (GHDF), as shown in Figure 4. All samples showed absorption bands between 400 and 4000 cm^−1^, which is where the signals of polysaccharide and cellulose structures from green rice husk, the main part of the product, may be found. At 3450–3400 and 3300 cm^−1^, a strong absorption band was seen. This was due to the O–H stretching vibrations of cellulose [6]. The absorption band at 2925 cm^−1^ was due to C–H stretching, which is usually linked to carbohydrate structures [53]. The bands around 1642 cm^−1^ and approximately 1200 cm^−1^ were also linked to O–H bending vibrations that had to do with water being absorbed by cellulose fibers [8]. Absorption bands between 1500 and 1400 cm^−1^ suggested the presence of phenolic compounds, which are commonly found in plant hulls [54]. The band at 1510 cm^−1^ was related to C–H bending in polysaccharides. The bands at 1037 and 1600 cm^−1^ were associated with C–O stretching, indicating the presence of glycosidic bonds between sugar units [25,33]. Additional bands between 900 and 800 cm^−1^ suggested β-glycosidic linkages among sugar units, which are typical of polysaccharide structures [55]. The absorption peaks observed at 640 and 538 cm^−1^ may be associated with C–O bending vibrations of carbonyl-containing groups, suggesting the presence of related linkages within the insoluble dietary fiber matrix [25]. When comparing samples with different levels of GHDF incorporation, the intensity of cellulose-related absorption bands at 3450, 2925, and 1642 cm^−1^ showed an increasing trend with higher fiber levels, suggesting enhanced contributions from cellulose-associated functional groups [6]. Regarding the effects of hydrocolloid addition, the type of hydrocolloid did not generate new peaks or cause peak shifts, aligning with the report of Liu et al. [13], which indicated that hydrocolloids do not form new covalent bonds with polysaccharide networks in similar systems However, differences in the structure of the gelling agents may change the strength of some FTIR signals, especially in the O–H region (3000–3600 cm^−1^), due to differences in hydrogen bonding in the gummies. Overall, the changes in the FTIR spectra were mainly related to the amount of dietary fiber in the gummies, which shows differences in the product composition. These results may help in developing gummy and other gel-based food products.

### 3.8. Bioactive Compounds and Antioxidant Activity

Bioactive compounds and antioxidant activity are key indicators that reflect the health-promoting potential of functional food products [56,57]. Table 4 shows that the amount of green rice husk dietary fiber (GHDF) and the type of hydrocolloids significantly affected the total phenolic content (TPC), total flavonoid content (TFC), and antioxidant activity measured by the DPPH and FRAP assays. The TPC and TFC values ranged from 11.02 to 35.17 mg GAE/100 g and 14.47 to 27.97 mg QE/100 g, respectively. As the level of GHDF increased, the levels of bioactive compounds and antioxidant activity also increased. Samples containing 5% GHDF generally showed higher values than those with 3% and 1% GHDF. The TPC of gummies fortified with green rice husk was approximately three times higher than that of gummies supplemented with Garcinia extract, as reported by Renaldi et al. [42], but it was still lower than that of the apple cider vinegar-enriched jellies reported by Yi et al. [38], likely due to differences in raw material composition and formulation. The DPPH and FRAP values for antioxidant activity were 59.92–67.74 mg AA/100 g and 2.66–10.30 mg FeSO4/100 g, respectively. These values were almost twice as high as those for Garcinia and soursop gummies [42,58]. The increasing trend in TPC, TFC, DPPH, and FRAP values with higher levels of green rice husk supplementation may be attributed to the phenolic compounds present in rice husk [7]. This observation is consistent with findings that green rice husk powder enhances phenolic content in meat products [59]. However, the measured levels of bioactive compounds in the final product may also be influenced by other components in the formulation. Apart from fiber concentration, the type of hydrocolloid also played a crucial role in determining the measured bioactive properties. Formulations containing CC and GG exhibited higher levels of bioactive compounds compared with those containing XG, likely due to differences in polymer structures and the resulting microstructural organization of the gel matrix [45,47]. CC forms a more brittle and porous network, which may facilitate the diffusion and distribution of bioactive compounds, whereas GG and XG produce more viscous matrices that can entrap compounds within the gel structure, potentially limiting their release [52]. These findings align with those of Aguirre Calvo et al. [57], who reported that GG improved the retention of betacyanin and polyphenols in alginate matrices. Nevertheless, variations in extraction conditions and analytical methods may also contribute to differences in detected bioactive levels. In conclusion, the incorporation of GHDF and the selection of appropriate hydrocolloids are critical determinants of the bioactive and antioxidant potential of gummy products. These results provide valuable insights for the formulation and development of future functional food applications.

### 3.9. Sensory Evaluation

**Figure 5:**
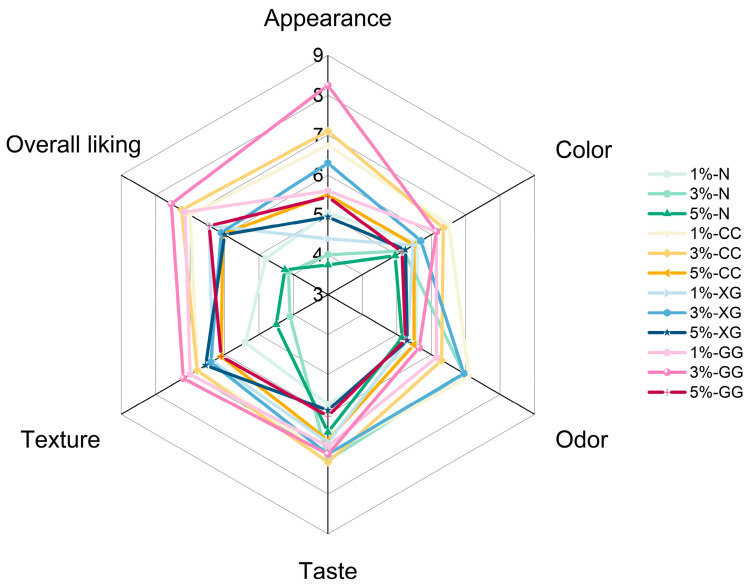
Sensory properties of green rice husk dietary fiber. The values 1%, 3%, and 5% represent the levels of added green rice husk dietary fiber (GDHF). N = no hydrocolloids added; XG = xanthan gum; CC = carrageenan; GG = guar gum. The sensory evaluation was conducted with 30 panelists.

Sensory evaluation is commonly used to assess consumer acceptance of food products by examining the perception of various sensory attributes, which plays an important role in product development [24]. Sensory evaluation of the gummy fortified with green rice husk dietary fiber (GDHF) was conducted using a 9-point hedonic scale to assess consumer acceptability in terms of appearance, color, aroma, taste, texture, and overall liking, as presented in Figure 5. Both the level of GHDF and the type of hydrocolloid significantly influenced the sensory acceptance scores. Samples without added hydrocolloids received lower liking scores than those containing hydrocolloids. Regarding color and aroma, the 1% CG formulation achieved the highest acceptability, whereas the 3% GG formulation received the highest scores for texture and overall liking. These findings indicate that the combination of dietary fiber and hydrocolloids plays a crucial role in shaping sensory attributes. Increasing the amount of dietary fiber resulted in significant changes in the product’s appearance, color, aroma, taste, texture, and overall acceptability. This can be attributed to the incorporation of fiber particles within the gel network, which affects the structural integrity and visual characteristics of the product [16]. Additionally, hydrocolloids contributed to improvements in textural properties by enhancing the strength and uniformity of the gel matrix [42]. Therefore, the sensory results suggest that variations in the level of dietary fiber and the type of hydrocolloid are associated with differences in sensory acceptance, likely through their combined influence on gel structure and texture [60,61], which are key factors in consumer perception.

However, the sensory acceptance assessment conducted in this research should be considered only a preliminary evaluation. Therefore, future studies are needed using larger consumer groups to confirm broader consumer acceptance. Furthermore, future research may include market-focused assessments, such as price perception and purchase intention, to better evaluate the commercial potential and sensory acceptance of products.

## 4. Conclusions

The incorporation of insoluble dietary fiber into food systems may influence processing characteristics, sensory properties, and the stability of the final product. In particular, inadequate dispersion of fiber particles may lead to sedimentation within the product matrix. This study explored the application of green rice husk dietary fiber (GDHF) in combination with hydrocolloids in gummy products, with the aim of evaluating their effects on physicochemical properties, textural characteristics, rheological behavior, bioactive compounds, and sensory acceptance. The results indicated that GHDF shows potential for enhancing nutritional value and bioactive compound content, as well as influencing the structural properties of the gummies. Meanwhile, hydrocolloids contributed to improvements in appearance, texture, rheological behavior, and trends in consumer sensory acceptance. The combined incorporation of GHDF and hydrocolloids increased the diversity of nutritional components in gummy products. This study shows that green rice husk, a by-product of the rice milling process, could be a long-lasting source of nutritional fiber for food products that add value. Nonetheless, certain limits must be recognized. This study did not look into the storage stability and shelf life of the items that were developed, and the relatively high water activity may make the products less stable while they are being stored. So, future studies should focus on testing how well GDHF remains stable in storage and improving its formulation to make it easier to use in food products. However, these early results could be used as a starting point for additional research and development of formulations that use GDHF.

## Figures and Tables

**Table 1:** Ingredient compositions of green rice husk gummy fiber formulations.

Formulations	Hydrocolloids (g/100 g)	Water (g/100 g)	Other Ingredients (g/100 g)
1%-N	-	55	Gelatin (8 g/100 g), Glucose syrup (10 g/100 g), Sugar (26 g/100 g), Citric acids (0.5 g/100 g)
1%-XG	0.5	54	
1%-CC	0.5	54	
1%-GG	0.5	54	
3%-N	-	55	
3%-XG	0.5	52	
3%-CC	0.5	52	
3%-GG	0.5	52	
5%-N	-	55	
5%-XG	0.5	50	
5%-CC	0.5	50	
5%-GG	0.5	50	

Adding the difference in the sample with dietary fiber will compensate for less water. The values 1%, 3%, and 5% represent the levels of added green rice husk dietary fiber (GDHF). N = no hydrocolloids added; XG = xanthan gum; CC = carrageenan; GG = guar gum.

**Table 2:** Color parameters, water activity (aw), pH value, and syneresis percentage of green rice husk gummy fiber.

Fiber	Hydrocolloids	Color			a_w_	pH Value ^ns^	Syneresis %
		L*	a*	b*			
1%	N	43.91 ± 0.02 ^b^	1.24 ± 0.05 ^b^	12.03 ± 0.06 ^a^	0.89 ± 0.02 ^a^	4.87 ± 0.01	6.44 ± 0.23 ^a^
	XG	46.06 ± 0.33 ^a^	1.59 ± 0.02 ^a^	10.29 ± 0.05 ^abc^	0.88 ± 0.01 ^ab^	4.87 ± 0.01	1.28 ± 0.01 ^d^
	CC	41.85 ± 0.02 ^c^	0.87 ± 0.07 ^c^	6.50 ± 5.19 ^d^	0.87 ± 0.01 ^ab^	4.87 ± 0.01	1.19 ± 0.01 ^f^
	GG	34.85 ± 0.27 ^h^	0.81 ± 0.02 ^c^	11.03 ± 0.59 ^ab^	0.88 ± 0.01 ^ab^	4.87 ± 0.01	1.20 ± 0.03 ^f^
3%	N	39.05 ± 0.01 ^d^	0.52 ± 0.02 ^e^	10.06 ± 0.05 ^abc^	0.88 ± 0.01 ^ab^	4.87 ± 0.01	5.73 ± 0.03 ^b^
	XG	35.91 ± 0.30 ^f^	0.38 ± 0.05 ^f^	9.49 ± 0.09 ^abc^	0.88 ± 0.01 ^ab^	4.87 ± 0.01	1.23 ± 0.01 ^e^
	CC	34.79 ± 0.19 ^h^	0.67 ± 0.02 ^d^	7.75 ± 0.18 ^cd^	0.87 ± 0.01 ^ab^	4.87 ± 0.01	1.13 ± 0.02 ^g^
	GG	34.66 ± 0.47 ^h^	0.73 ± 0.05 ^d^	7.68 ± 0.18 ^cd^	0.88 ± 0.01 ^ab^	4.87 ± 0.01	1.14 ± 0.01 ^g^
5%	N	35.29 ± 0.03 ^g^	0.04 ± 0.01 ^h^	10.14 ± 0.04 ^abc^	0.88 ± 0.01 ^ab^	4.87 ± 0.01	3.12 ± 0.10 ^c^
	XG	36.77 ± 0.11 ^e^	0.05 ± 0.04 ^h^	8.00 ± 0.06 ^cd^	0.87 ± 0.01 ^ab^	4.87 ± 0.01	1.14 ± 0.02 ^g^
	CC	34.69 ± 0.49 ^h^	0.12 ± 0.02 ^g^	8.52 ± 0.16 ^bcd^	0.88 ± 0.01 ^ab^	4.87 ± 0.01	1.08 ± 0.01 ^h^
	GG	31.20 ± 0.18 ^i^	0.30 ± 0.06 ^f^	8.36 ± 0.14 ^bcd^	0.87 ± 0.01 ^b^	4.87 ± 0.01	1.03 ± 0.02 ^h^

The values are presented as mean ± standard deviation (*n* = 3). Superscripts ^a–i^ indicate that the means in the same column are significantly different (*p* < 0.05), while ^ns^ denotes no significant difference (*p* > 0.05). The values 1%, 3%, and 5% represent the levels of added green rice husk dietary fiber (GDHF). N = no hydrocolloids added; XG = xanthan gum; CC = carrageenan; GG = guar gum.

**Table 3:** Proximate composition of green rice husk gummy fiber (g/100 g FW).

GHDF	Hydrocolloids	Moisture	Proteins	Carbohydrate	Fiber	Lipids ^ns^	Ash
1%	N	47.71 ± 0.05 ^a^	6.83 ± 0.06 ^bcd^	43.89 ± 0.07 ^abc^	1.03 ± 0.06 ^d^	0.20 ± 0.02	0.33 ± 0.01 ^c^
	XG	47.64 ± 0.03 ^b^	7.43 ± 0.23 ^a^	43.47 ± 0.23 ^cd^	1.01 ± 0.02 ^d^	0.17 ± 0.03	0.27 ± 0.04 ^cd^
	CC	47.72 ± 0.01 ^a^	7.10 ± 0.26 ^ab^	43.69 ± 0.32 ^abc^	1.00 ± 0.01 ^d^	0.18 ± 0.04	0.31 ± 0.01 ^cd^
	GG	47.71 ± 0.02 ^a^	6.90 ± 0.20 ^bc^	43.88 ± 0.18 ^abc^	1.02 ± 0.01 ^d^	0.21 ± 0.08	0.29 ± 0.02 ^cd^
3%	N	45.98 ± 0.01 ^c^	6.73 ± 0.11 ^bcd^	43.72 ± 0.09 ^abc^	2.96 ± 0.03 ^c^	0.18 ± 0.02	0.42 ± 0.04 ^b^
	XG	45.99 ± 0.01 ^c^	6.47 ± 0.12 ^de^	44.03 ± 0.17 ^ab^	2.97 ± 0.03 ^c^	0.16 ± 0.02	0.40 ± 0.01 ^b^
	CC	45.95 ± 0.03 ^c^	6.23 ± 0.49 ^e^	44.21 ± 0.44 ^a^	2.99 ± 0.01 ^c^	0.20 ± 0.03	0.41 ± 0.02 ^b^
	GG	46.03 ± 0.01 ^c^	6.6 ± 0.20 ^cde^	43.82 ± 0.26 ^abc^	2.99 ± 0.02 ^c^	0.19 ± 0.02	0.39 ± 0.02 ^b^
5%	N	44.38 ± 0.04 ^e^	6.83 ± 0.25 ^bcd^	43.05 ± 0.24 ^ef^	4.97 ± 0.03 ^ab^	0.21 ± 0.01	0.56 ± 0.03 ^a^
	XG	44.46 ± 0.07 ^d^	7.00 ± 0.01 ^bc^	42.81 ± 0.13 ^f^	5.02 ± 0.02 ^a^	0.17 ± 0.04	0.54 ± 0.04 ^a^
	CC	44.39 ± 0.01 ^e^	6.67 ± 0.12 ^cd^	43.23 ± 0.14 ^de^	4.97 ± 0.04 ^ab^	0.19 ± 0.03	0.56 ± 0.03 ^a^
	GG	44.39 ± 0.02 ^e^	6.93 ± 0.15 ^bc^	42.98 ± 0.08 ^ef^	4.95 ± 0.06 ^b^	0.19 ± 0.02	0.55 ± 0.01 ^a^

The values are presented as mean ± standard deviation (*n* = 3). Superscripts ^a–f^ indicate that the means in the same column are significantly different (*p* < 0.05), while ^ns^ denotes no significant difference (*p* > 0.05). The values 1%, 3%, and 5% represent the levels of added green rice husk dietary fiber (GDHF). N = no hydrocolloids added; XG = xanthan gum; CC = carrageenan; GG = guar gum.

**Table 4:** Total phenolic content (TPC), total flavonoid content (TFC), and antioxidant activities (FRAP and DPPH) of green rice husk gummy fibers.

GHDF	Hydrocolloids	TPC (mg GAE/100 g FW)	TFC (mg QE/100 g FW)	FRAP (mg FeSO_4_/100 g FW)	DPPH (mg AA/100 g FW)
1%	N	11.40 ± 0.37 ^d^	14.67 ± 0.66 ^e^	2.76 ± 0.39 ^e^	53.47 ± 0.30 ^d^
	XG	11.02 ± 0.14 ^d^	14.47 ± 0.68 ^e^	2.66 ± 0.12 ^e^	49.92 ± 0.20 ^g^
	CC	11.62 ± 0.21 ^d^	15.48 ± 0.37 ^e^	2.91 ± 0.41 ^e^	51.13 ± 0.29 ^e^
	GG	11.87 ± 0.11 ^d^	14.59 ± 0.37 ^e^	3.01 ± 0.29 ^d^	50.34 ± 0.26 ^g^
3%	N	24.86 ± 0.58 ^c^	21.12 ± 0.38 ^c^	7.76 ± 0.12 ^c^	63.12 ± 0.30 ^d^
	XG	23.80 ± 0.57 ^c^	20.03 ± 0.36 ^d^	7.36 ± 0.49 ^c^	60.76 ± 0.29 ^f^
	CC	24.01 ± 1.18 ^c^	20.68 ± 0.99 ^dc^	7.83 ± 0.19 ^c^	62.02 ± 0.28 ^e^
	GG	24.26 ± 0.78 ^c^	20.21 ± 0.64 ^cd^	7.51 ± 0.12 ^c^	61.89 ± 0.29 ^e^
5%	N	35.06 ± 0.18 ^a^	27.97 ± 0.68 ^a^	10.30 ± 0.44 ^a^	67.74 ± 0.06 ^b^
	XG	33.90 ± 0.54 ^b^	25.89 ± 0.37 ^b^	9.37 ± 0.11 ^b^	65.92 ± 0.28 ^c^
	CC	35.17 ± 1.32 ^a^	27.92 ± 0.39 ^a^	10.13 ± 0.24 ^a^	66.84 ± 0.57 ^c^
	GG	34.24 ± 0.28 ^ab^	26.09 ± 0.65 ^b^	9.98 ± 0.301 ^a^	67.32 ± 0.59 ^ab^

The values are presented as mean ± standard deviation (*n* = 3). Superscripts ^a–g^ indicate that the means in the same column are significantly different (*p* < 0.05). The values 1%, 3%, and 5% represent the levels of added green rice husk dietary fiber (GDHF). N = no hydrocolloids added; XG = xanthan gum; CC = carrageenan; GG = guar gum.

## Data Availability

The original contributions presented in this study are included in the article. Further inquiries can be directed to the corresponding author.
